# Phytocyanin-encoding genes confer enhanced ozone tolerance in *Arabidopsis thaliana*

**DOI:** 10.1038/s41598-022-25706-0

**Published:** 2022-12-22

**Authors:** Shoko Saji, Hikaru Saji, Kimiyo Sage-Ono, Michiyuki Ono, Nobuyoshi Nakajima, Mitsuko Aono

**Affiliations:** 1 Biodiversity Division, National Institute for Environmental Studies, 16-2 Onogawa, Tsukuba, 305-8506 Japan; 2 Graduate School of Life and Environmental Science, University of Tsukuba, 1-1-1 Tennodai, Tsukuba, 305-8572 Japan

**Keywords:** Plant cell biology, Plant physiology, Plant stress responses, Environmental impact, Plant sciences, Environmental sciences

## Abstract

Ozone is a phytotoxic air pollutant that has various damaging effects on plants, including chlorosis and growth inhibition. Although various physiological and genetic studies have elucidated some of the mechanisms underlying plant ozone sensitivity and lesion development, our understanding of plant response to this gas remains incomplete. Here, we show evidence for the involvement of certain apoplastic proteins called phytocyanins, such as AtUC5, that protect against ozone damage. Two representative ozone-inducible responses, chlorosis and stomatal closure, were suppressed in AtUC5-overexpressing plants. Analysis of transgenic plants expressing a chimeric protein composed of AtUC5 fused to green fluorescent protein indicated that this fusion protein localises to the apoplast of plant cells where it appears to suppress early responses to ozone damage such as generation or signalling of reactive oxygen species. Moreover, yeast two-hybrid analyses suggest that AtUC5 may physically interact with stress-related proteins such as copper amine oxidase and late embryogenesis abundant protein-like protein. In addition to AtUC5, other examined phytocyanins such as AtUC6 and AtSC3 could confer ozone tolerance to plants when overexpressed in *A. thaliana*, suggesting that these proteins act together to protect plants against oxidative stress factors.

## Introduction

Tropospheric ozone (O_3_) is a phytotoxic air pollutant whose global background concentration has more than doubled over the past century. Moreover, tropospheric O_3_ concentration is predicted to increase even further over the next century in rapidly industrializing regions, posing a serious threat to both ecosystems and agriculture^1,2^. Therefore, it is necessary to elucidate the mechanisms of damage and protective responses of plants to this gas and utilize this knowledge to adjust to these changing circumstances.

The effects of O_3_ on plants are often characterised as either acute or chronic^3,4^. Acute exposure of plants to high concentrations of O_3,_ e.g*.* 0.2 µL L^−1^ for durations ranging from a few hours to a few days induces chlorotic foliar lesions, the result of mesophyll cell death in O_3_-sensitive plants. Chronic exposure to lower levels of the pollutant for several days or weeks reduces the growth rate and hastens the onset of senescence. Various physiological, biochemical, and genetic studies have uncovered some of the mechanisms of plant O_3_ sensitivity and O_3_ lesion development. O_3_ and the reactive oxygen species (ROS) that result from O_3_ degradation in the apoplast can either cause direct oxidative damage or induce programmed cell death by activating several signal transduction pathways similar to those induced by pathogen infection^5,6^.

Plants have various protective mechanisms against O_3_ damage which have been uncovered mainly by genetic studies using either *Arabidopsis thaliana* mutants or accessions that exhibit different O_3_ sensitivities. Since the rate of O_3_ absorption by plants is governed by the degree of stomatal aperture, mutants^7,8^ or ecotypes^9,10^ with higher stomatal conductance have been found to be highly sensitive to O_3_. Another group of O_3_-sensitive mutants comprises those with defects in either the antioxidative^11,12^ or photorespiratory pathway^13^, which indicates that maintaining ROS or reducing equivalents at low levels is essential for protection against O_3_. The third group consists of mutants with altered levels of, or responsiveness to either ethylene, salicylic acid, jasmonic acid, or nitric oxide, which suggests that these signalling compounds are involved in the plant’s response to O_3_^14–17^. Additionally, heterotrimeric G protein subunits and NADPH oxidase knockout mutants^18^, along with transgenic plants with altered expression of genes involved in mitogen-activated protein kinase cascades^19–21^, have been reported to exhibit altered O_3_ responses.

Despite the considerable amount of information obtained from these molecular studies, our understanding of plant response to O_3_ remains incomplete. For example, little is known about the early steps of O_3_ responses that occur in the extracellular space when either O_3_ or ROS resulting from its degradation oxidise various biomolecules and trigger intracellular as well as self-propagating systemic signals^22,23^. In the study described here, we report the isolation of an O_3_-tolerant *A. thaliana* line that overexpresses a member of the phytocyanin group which functions in the apoplast of plant cells. We expect that investigations of this and other transgenic lines of plants that we have generated will provide novel insights into the regulating mechanisms of O_3_-induced early signals. These investigations are also expected to contribute to the generation of O_3_-tolerant lines of crops or trees.

## Results

### Isolation of an O_3_-tolerant *A. thaliana* line and identification of the mutation responsible for the O_3_ tolerance

To isolate O_3_-tolerant plants, we grew seedlings of full-length cDNA overexpressor (FOX) lines of *A. thaliana*, ecotype Col-0, under long-day conditions of 14 h exposure to 100 µmol m^−2^ s^−1^ illumination and 10 h of darkness for 2 weeks. Next, we exposed them to 0.3 µL L^−1^ O_3_ under 420 µmol m^−2^ s^−1^ irradiation for a few hours. Clear chlorotic symptoms were observed in these seedlings on the following day. We identified a plant with few symptoms among the heavily damaged seedlings grown from approximately 3000 seeds from the FOX lines (Supplementary Fig. 1).

**Figure 1:**
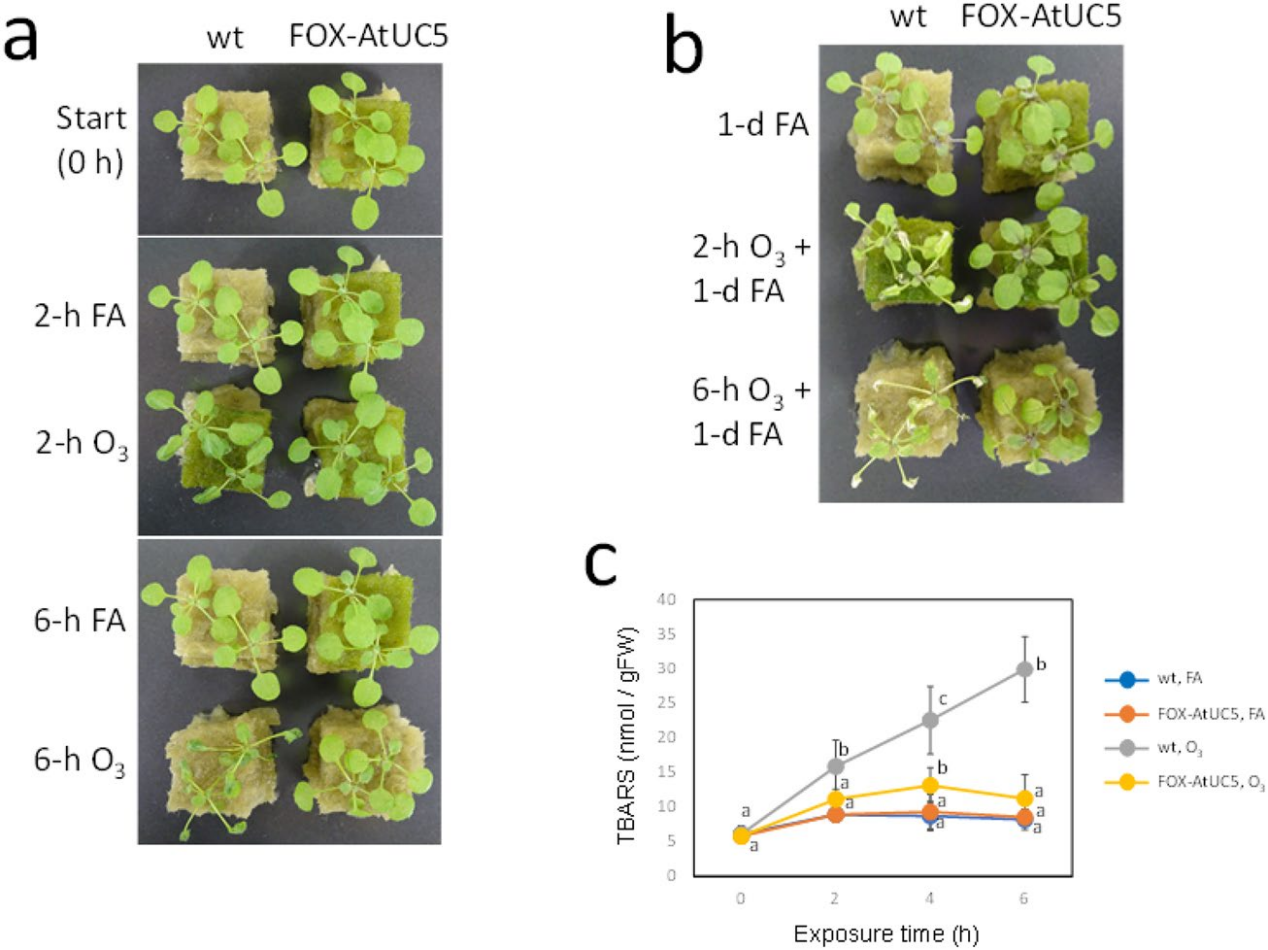
O_3_-tolerant phenotypes of FOX-AtUC5 plants. (**a**) Images of wild-type and FOX-AtUC5 plants exposed for 0, 2, or 6 h to fresh air or 0.3 µL L^−1^ O_3_ under 420 µmol photons m^−2^ s^−1^ illumination. (**b**) Images of these plants kept for another day in fresh air under a continuous illumination at 420 µmol photons m^−2^ s^−1^. (**c**) Foliar content of thiobarbituric acid-reactive substances (TBARS) measured at various hours after the onset of exposure of wild-type and FOX-AtUC5 plants to fresh air or 0.3 µL L^−1^ O_3_ under continuous illumination with 420 μmol photons m^−2^ s^−1^. Mean and SD are shown (*n* = 9). At each time, means with the same letter are not significantly different from each other at *p* < 0.05. *wt* wild-type, *FA* fresh air, *TBARS* thiobarbituric acid-reactive substances.

PCR amplification from DNA extracted from the isolated plant using primers across the vector’s multiple cloning site yielded a roughly 1300 bp DNA fragment (Supplementary Fig. 2). The nucleotide sequence of this PCR product indicated that it corresponds to the cDNA for the gene *At1g72230* or “*AtUC5*” designated by Ma et al.^24^ which encodes a phytocyanin, specifically an uclacyanin^25^. The cDNA encodes a N-terminal signal peptide, a copper-binding plastocyanin-like domain, an arabinogalactan protein-like region, and a C-terminal glycosylphosphatidylinositol (GPI) attachment signal. Following line purification by backcrossing against the wild-type plant, we generated a plant homozygous for this cDNA and named this plant line “FOX-AtUC5”. While two-week-old seedlings of wild-type plants exposed to 0.3 µL L^−1^ O_3_ begin to wither as early as 2 h after the start of O_3_ treatment and exhibit severe chlorosis on the next day, such visible damage was not observed at all in FOX-AtUC5 under the same conditions (Fig. 1a,b). FOX-AtUC5 also exhibited enhanced O_3_ tolerance in the foliar content of thiobarbituric acid-reactive substances that is a marker for lipid peroxidation and oxidative tissue injury^26^ (Fig. 1c).

According to available information^27,28^, AtUC5 is thought to localise to the apoplast (plasma membrane or cell wall) of various organs, including leaves. Although the physiological functions of AtUC5 are unknown, some other phytocyanins are reportedly involved in various aspects of growth and development as well as stress responses. To confirm that AtUC5 overproduction is responsible for the enhanced O_3_ tolerance of FOX-AtUC5 plants, we independently generated *AtUC5-*overexpressing *A. thaliana* for two ecotypes, Col-0 and Ws-2. Plants expressing this protein at various levels were generated and a correlation between the level of *AtUC5* expression and O_3_ tolerance was observed in these plants with two different background ecotypes (Supplementary Fig. 3). These results support the conclusion that AtUC5 overexpression is responsible for enhanced O_3_ tolerance in FOX-AtUC5 and that *AtUC5* overexpression can enhance O_3_ tolerance in at least two different *A. thaliana* ecotypes.

We also obtained seeds of an *AtUC5* knockout mutant and determined its O_3_ sensitivity. However, we did not observe clear differences between this mutant and wild-type plants, at least under the conditions used in our study (Supplementary Fig. 4).

### Attenuation of stomatal response towards O_3_ in FOX-AtUC5 plants

**Figure 2:**
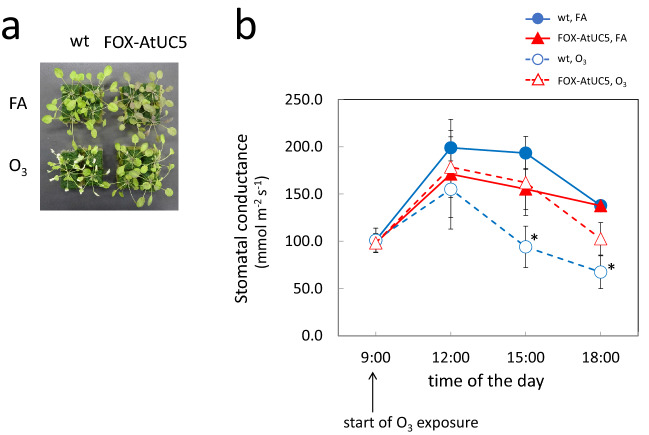
Foliar and stomatal response to O_3_ of AtUC5 grown under a short-day condition. (**a**) Images of wild-type and FOX-AtUC5 plants grown under a condition of 8-h light at 100 µmol m^−2^ s^−1^/16-h dark for 5 weeks and exposed for 8 h to fresh air or 0.3 µL L^−1^ O_3_ under 420 µmol photons m^−2^ s^−1^ illumination, followed by a further exposure to fresh air for 1 day. (**b**) Stomatal conductances of wild-type and FOX-AtUC5 plants grown and treated in these ways, measured at various hours after the start of O_3_ exposure. The averages and SD are shown (*n* = 5); asterisks indicate significantly different values from those of the wild-type plants at each timepoint exposed to fresh air (*p* < 0.05). *wt* wild type, *FA* fresh air.

As described in “Introduction”, the different O_3_ sensitivities in some *A. thaliana* accessions are due to differences in stomatal response that cause different O_3_ absorption rates. Therefore, we investigated the stomatal response of FOX-AtUC5 plants. To do so, we grew FOX-AtUC5 and wild-type plants under short-day conditions for 5 weeks until their leaves were big enough for stomatal conductance to be measured with a leaf porometer. After verifying that there is a clear difference in O_3_ sensitivity between FOX-AtUC5 and wild-type plants grown in this way (Fig. 2a), we measured stomatal conductance for water vapour of the leaves of plants grown in this way, either treated with O_3_ or untreated, at various timepoints following O_3_ exposure. The stomatal conductance showed diurnal variation, increasing in the morning and decreasing in the afternoon. There was no significant difference in stomatal conductance between FOX-AtUC5 and wild-type plants in the absence of O_3_ (Fig. 2b). While the stomatal conductance of wild-type plants was lowered by O_3_ exposure as previously reported ^7,13^, this effect was not observed in FOX-AtUC5 plants. Since the stomatal conductance of FOX-AtUC5 plants was always either higher than or similar to that of wild-type plant, it is unlikely that stomatal involvement is the causal factor for O_3_ tolerance in FOX-AtUC5 plants. Furthermore, both of the O_3_-inducible responses—stomatal closure and visual damage due to foliar cell death—were shown to be suppressed in FOX-AtUC5 plants. These responses operate at different sites on leaves, specifically guard cells and mesophyll cells, respectively.

### AtUC5 localises to and functions in the apoplast of plant cells

**Figure 3:**
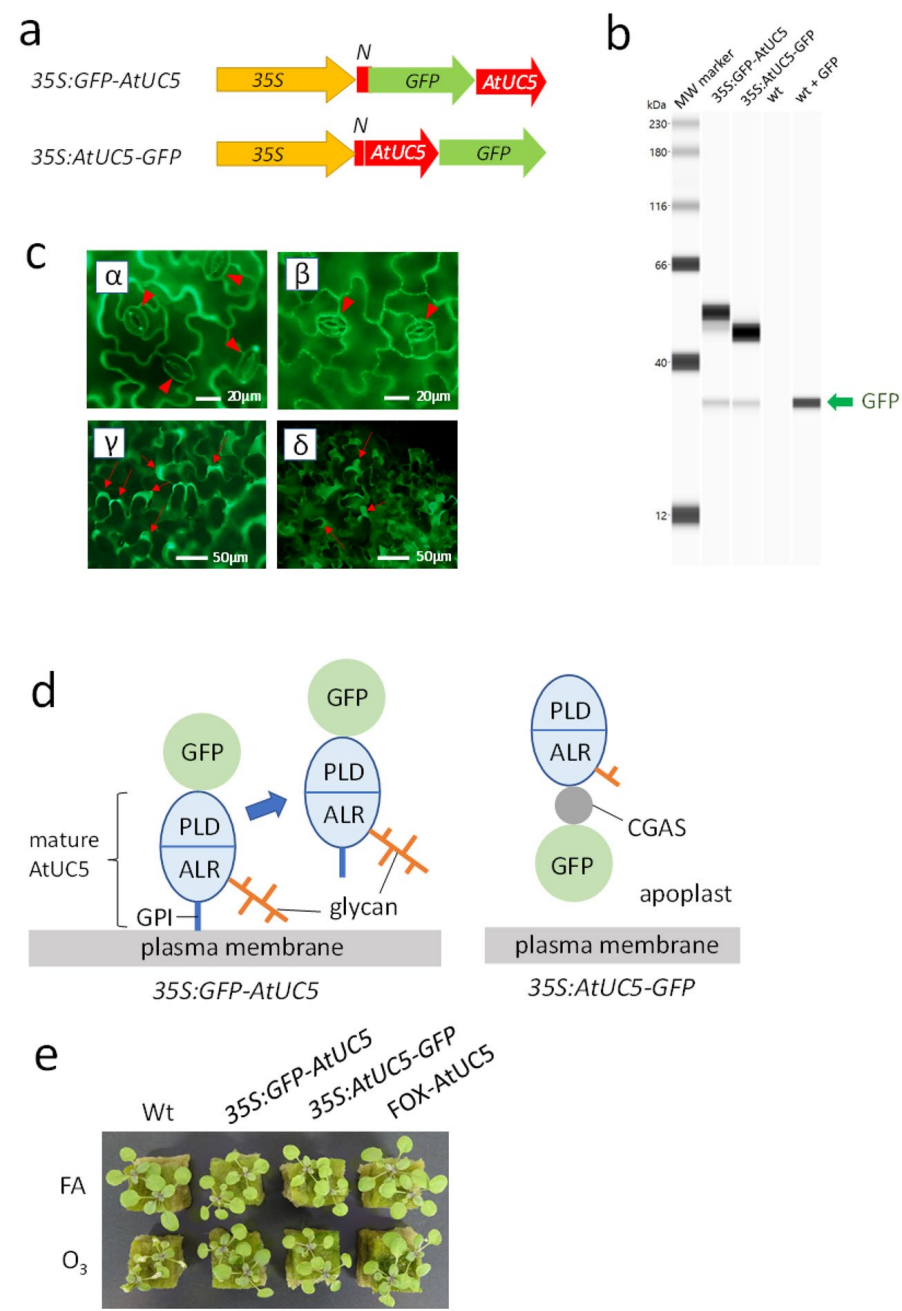
Cellular localisation and the ability of AtUC5-GFP fusion proteins to confer O_3_ tolerance. (**a**) Diagrams depicting the chimeric genes to produce fusion proteins in transgenic plants. (**b**) Results of western blotting to detect fusion protein expression in extracts of transgenic plants. Equal amounts (3 µg) of protein extracted from plants of various lines were subjected to electrophoresis and probed with anti-GFP antibody using Simple Western™ (Bio-Techne, Minneapolis, USA). GFP was added to the protein extract from the wild-type plant just before the electrophoresis for the lane “wt + GFP.” Results were obtained as digital data for each sample and exhibited as band images as their full-length blots. (**c**) Microscopic observation of GFP fluorescence in epidermal tissues of transgenic plants. *α* fluorescence images of epidermal tissue of a leaf transgenic for *35S:GFP-AtUC5*. *β* fluorescence image of epidermal tissue of a leaf transgenic for *35S:AtUC5-GFP*. *γ* fluorescence image of epidermal tissue of a leaf transgenic for *35S:GFP-AtUC5* after treatment with hyperosmotic solution. *δ* fluorescence image of the epidermal tissue of a leaf transgenic for *35S:AtUC5-GFP* after treatment with hyperosmotic solution. Arrowheads and arrows denote stomata and apoplastic matrices, respectively. (**d**) Diagrams depicting the presumed structures and localizations of fusion proteins in transgenic plants. In *35S:GFP-AtUC5* plants, the fusion protein is released from the plasma membrane after digestion at the GPI moiety by phospholipases. (**e**) Images of various lines 1 day after 2 h exposure to fresh air or 0.3 µL L^−1^ O_3_ under 420 µmol photons m^−2^ s^−1^ illumination. *Wt *wild-type plants, *GFP* green fluorescent protein, *35S:GFP-AtUC5* *35S:AtUC5-GFP*, transgenic plants producing fusion proteins, *35S* cauliflower mosaic virus 35S promoter, *N* a fragment encoding the N-terminal signal peptide of AtUC5, *PLD* plastocyanin-like domain, *ALR* arabinogalactan protein-like region, *GPI* glycosylphosphatidylinositol moiety, *CGAS* C-terminal glycosylphosphatidylinositol-anchored signal, *FA* fresh air*.*

To perform histochemical analyses, we generated transgenic plants that produce chimeric proteins composed of AtUC5 fused to green-fluorescent protein (GFP). Two different DNA constructs were generated for the transformation. One of the two, *35S:GFP-AtUC5*, harbours a chimeric gene in which the GFP-encoding sequence is inserted between the DNA fragment encoding an N-terminal secretion signal and the DNA fragment encoding the plastocyanin-like domain of AtUC5 (Fig. 3a). In the other construct, *35S:AtUC5-GFP*, the GFP-encoding sequence was inserted after the sequence for the C-terminal GPI attachment signal of AtUC5. Expression of these constructs in transgenic plants should produce fusion proteins in which GFP is attached either to the N-terminus of mature AtUC5 or to the C-terminal GPI attachment signal which has not been removed by processing, respectively (Fig. 3d).

Some of the transgenic plants generated using both constructs exhibited enhanced O_3_ tolerance (Fig. 3e), indicating that the AtUC5 moiety of these fusion proteins can still confer O_3_ tolerance. We could detect GFP fusion proteins in extracts from these plants by western blotting using an anti-GFP antibody (Fig. 3b). We observed an approximately 53 kDa band corresponding to the fusion protein in the *35S:GFP-AtUC5* plant, which is 13 kDa more than the summed molecular masses of GFP and the mature AtUC5 polypeptide. The higher apparent molecular mass may be due to post-translational glycosylation at the arabinogalactan protein-like region of AtUC5 (Fig. 3d). In contrast, the fusion protein in the *35S:AtUC5-GFP* plant had an apparent mass of 48 kDa, only 5 kDa more than the molecular mass of the predicted fusion polypeptide. Therefore, the postulated glycosylation of the fusion protein of *35S:AtUC5-GFP* plant may either be incomplete or different from that of the fusion protein in the *35S:GFP-AtUC5* plant.

Microscopic observation of GFP fluorescence in transgenic plant epidermal tissues revealed the presence of fusion proteins at the peripheral region of epidermal cells, including stomatal guard cells, which is consistent with our expectations (Fig. 3c (α, β)). After the treatment of these leaves with a hyperosmotic solution, fluorescence was observed in the region between the plasma membrane and cell wall of plasmolysed cells (Fig. 3c (γ, δ)), which suggests that the fusion protein localises to apoplastic matrices. This likely occurs after digestion by a phospholipase at the GPI moiety in the *35S:GFP-AtUC5* plant (Fig. 3d). Since some transgenic plants generated with the two different constructs exhibited enhanced O_3_ tolerance (Fig. 3e), neither linkage to plasma membrane via GPI nor complete glycosylation appear necessary for AtUC5’s ability to confer O_3_ tolerance.

### AtUC5 may interact with stress-related proteins at the apoplastic region

**Figure 4:**
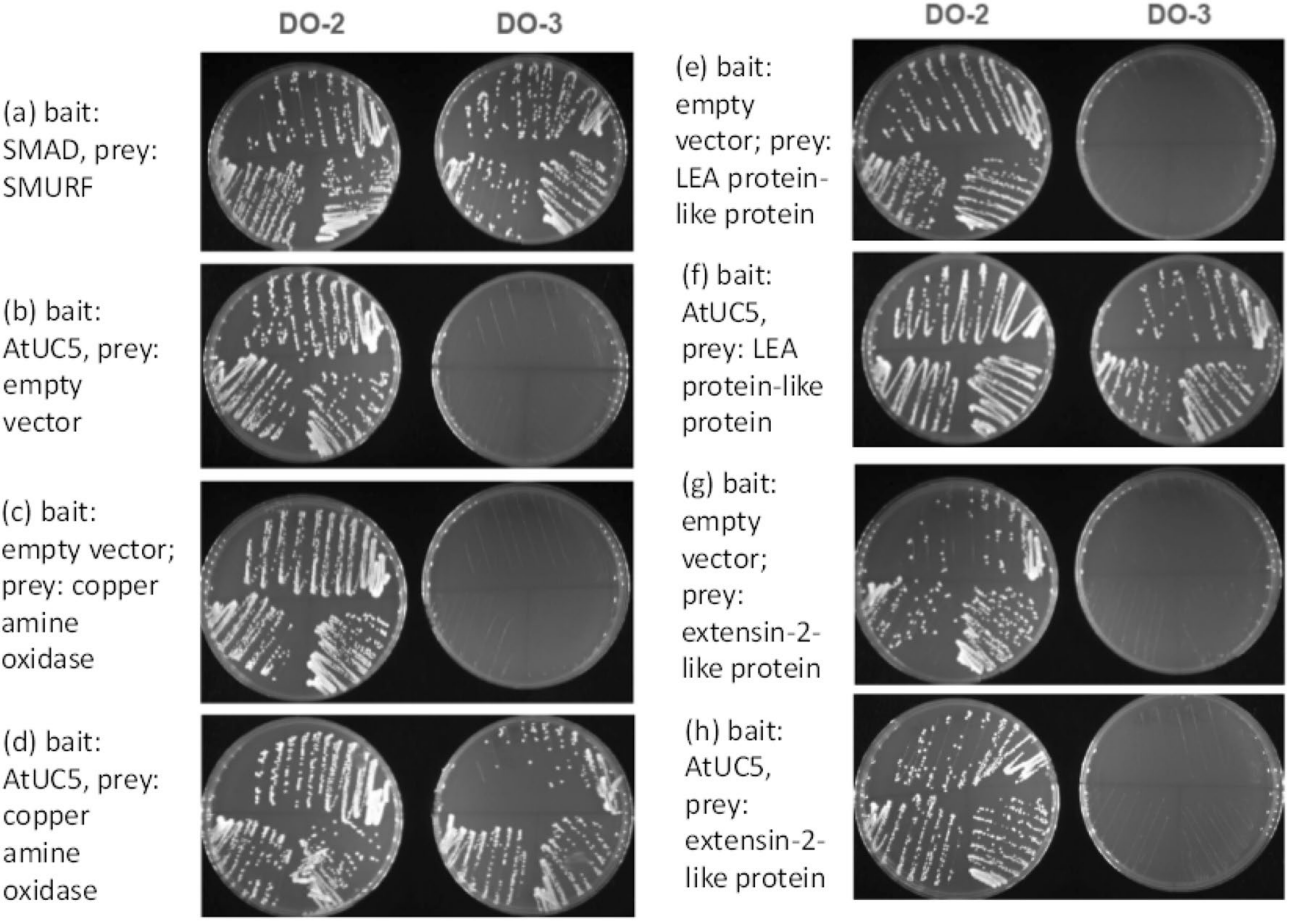
Characterising protein–protein interactions between AtUC5 and various proteins by pairwise yeast two-hybrid analysis. DO-2 indicates media without tryptophan and leucine, which selects for yeast containing both bait and prey plasmids. DO-3 indicates media without tryptophan, leucine, and histidine, which selects for yeast containing both bait and prey plasmids, and a protein–protein interaction between the bait and prey proteins. (**a**) Positive control (bait: SMAD, prey: SMURF). (**b**) Negative control (bait: AtUC5, prey: empty vector). (**c**) Negative control (bait: empty vector; prey: copper amine oxidase). (**d**) Protein–protein interaction (bait: AtUC5, prey: copper amine oxidase). (**e**) Negative control (bait: empty vector; prey: LEA protein-like protein). (**f**) Protein–protein interaction (bait: AtUC5, prey: LEA protein-like protein). (**g**) Negative control (bait: empty vector; prey: extensin-2-like protein). (**h**) Protein–protein interaction (bait: AtUC5, prey: extensin-2-like protein). SMAD, SMURF: interacting proteins in the human TGF-β/Smad pathway.

To clarify the function of AtUC5, we searched for interacting proteins using yeast two-hybrid analyses. First, we screened a prey cDNA library constructed from 1-week-old seedlings of *A. thaliana* using the coding sequence for the putative mature AtUC5 as bait; this coding sequence specifies a plastocyanin-like and an arabinogalactan-like domains. Among the 27 different polypeptides identified as putative interacting proteins in this screen, three have N-terminal signal peptides and are predicted to localise to the apoplast just like AtUC5 (Supplementary Fig. 5). The predicted amino acid sequences revealed that these polypeptides correspond to those of copper amine oxidase (AtCuAOγ2, At3g43670), late embryogenesis abundant (LEA) protein-like protein (At5g54370), and extensin-2-like protein, all of which are related to stress response. We tried to verify their interaction with AtUC5 by pairwise yeast two-hybrid analysis. Positive results were obtained with copper amine oxidase and LEA protein-like protein but not with extensin-2-like protein (Fig. 4). Therefore, AtUC5 may physically interact with the former two stress-related proteins within the apoplast of plant cells.

### Conferral of O_3_ tolerance by phytocyanins other than AtUC5

**Figure 5:**
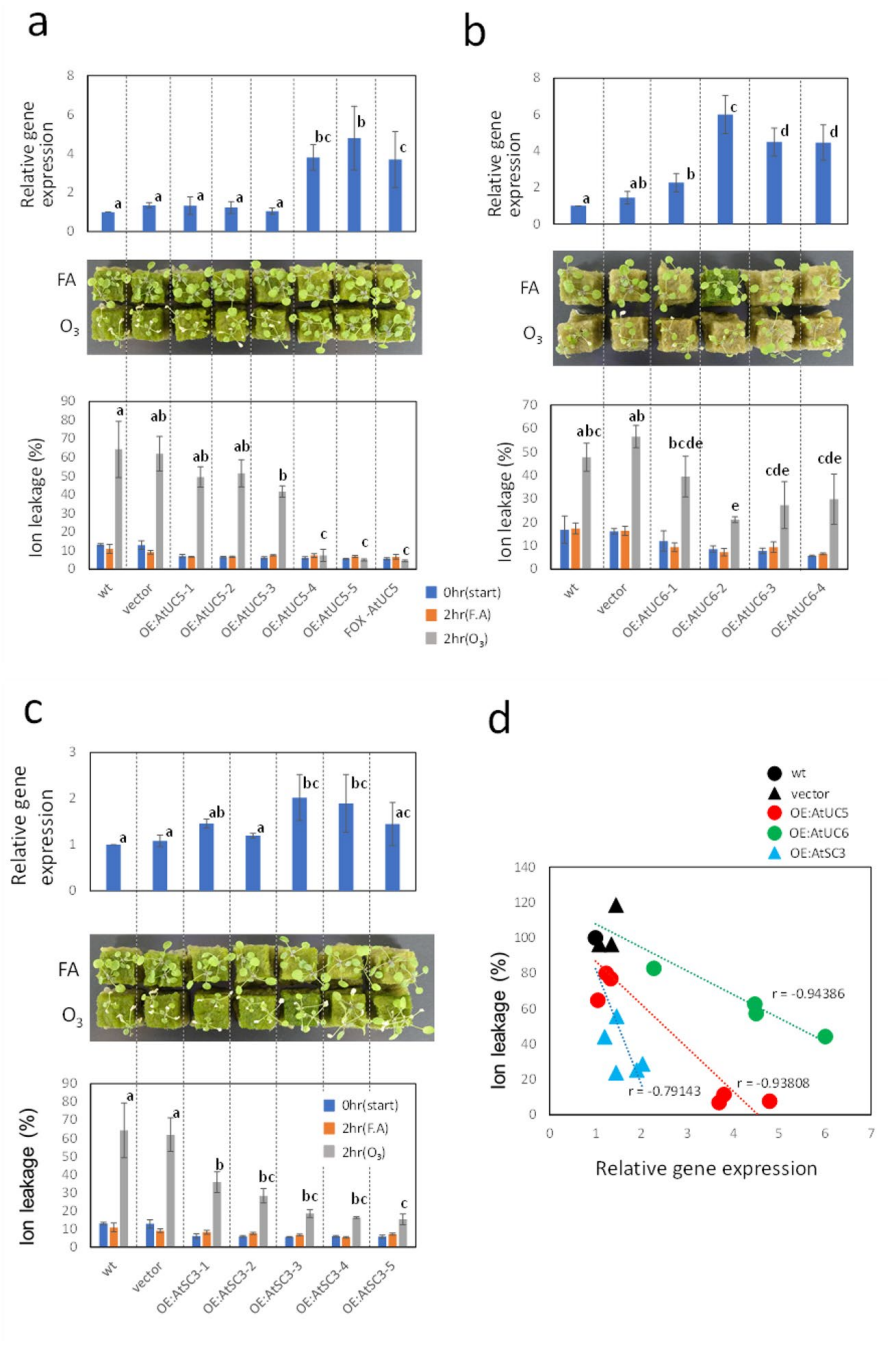
Correlations between transgene expression level and O_3_ tolerance observed in various *Arabidopsis* lines overexpressing different phytocyanin genes. (**a**–**c**) The levels of mRNA encoding *AtUC5* (**a**), *AtUC6* (**b**), and *AtSC3* (**c**) extracted from 2-week-old seedlings of each transgenic line were measured using reverse transcription PCR and relative values against the control plants (wt) are shown in the upper figure. Images of various plant lines 1 day after 2 h exposure to fresh air or 0.3 µL L^−1^ O_3_ under 420 µmol photons m^−2^ s^−1^ illumination are shown in the middle figure. The amounts of ion leakage from the leaves of plants that received either 0 or 2 h fresh air or 0.3 µL L^−1^ O_3_ under an irradiation at 420 µmol photons m^−2^ s^−1^ are shown in the bottom figure. Line names shown in the bottom figure also represent those of the upper and middle figures just above the bottom results. For the upper and bottom results, the mean and SD are shown (*n* = 3–16) and means with the same letter are not significantly different from each other at p < 0.05. *FA* fresh air, *wt* wild type, *vector* a plant to which only vector was introduced, *OE:AtUC5-1(or 2, 3, 4, 5)* *AtUC5*-overexpressing lines, *OE:AtUC6-1(or 2, 3, 4)* *AtUC6-*overexpressing lines; AtSC3-1 (or 2, 3, 4, 5), *AtSC3*-overexpressing lines. (**d**) Correlations between levels of transgene expression and ion leakage from leaves treated with ozone for 2 h, in various transgenic lines. The correlation coefficient (r) of the linear relationship for each transgenic line is shown in the figure.

Phytocyanins form a multigene family and the *A. thaliana* genome contains about 30 phytocyanin genes. To investigate whether other phytocyanins can also confer O_3_ tolerance, we generated transgenic plants that overexpress other phytocyanins. We chose two *A. thaliana* phytocyanin paralogs for this study: *AtUC6* (*AT3g27200*), which resembles *AtUC5* in structure and expression^24^; and *AtSC3* (*At5g20230*), which is reportedly involved in several stress responses as described in the Discussion. Transgenic plants that express these genes at various levels were obtained and their O_3_ sensitivities were determined by measuring the degree of chlorosis and ion leakage from leaf tissue. The results from these transgenic plants, as well as *AtUC5*-overexpressing plants, are shown in Fig. 5a–c and the correlations between levels of transgene expression and O_3_ sensitivities (levels of ion leakage from leaves after O_3_ treatment for 2 h) of all the transgenic lines are shown in Fig. 5d. These results indicate that not only *AtUC5*, but also other phytocyanin genes can confer O_3_ tolerance to plants, although the effectiveness is different between these transgenes.

## Discussion

By using FOX lines, we found a novel gene that may be involved in the plant response to O_3_. Therefore, FOX lines appear to be useful materials to study plant responses, in addition to knockout mutants which have been used in many studies. In FOX lines, a gene is over- or ectopically expressed and may exhibit phenotypes that are contrary to those of knockout or knockdown mutants in which the gene expression is suppressed. However, we did not observe higher O_3_ sensitivity in the knockout line for *AtUC5* (Supplementary Fig. 4). The reason for this is not clear but the redundant functions of genes may be responsible for this lack of phenotype in O_3_ response in the knockout line. Indeed, other phytocyanins such as AtUC6 and AtSC3 can also confer O_3_ tolerance and would be able to compensate for the lack of AtUC5. This may be the reason why phytocyanins have not been found in the studies screening O_3_-sensitive knockout or knockdown mutants.

**Figure 6:**
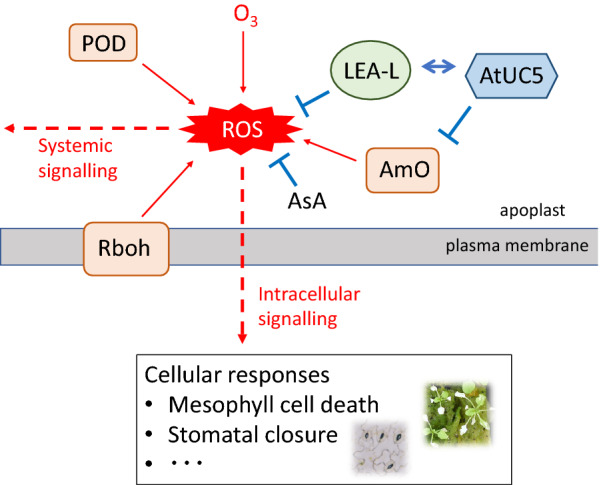
ROS generation/signalling and hypothetical acting sites of AtUC5 at the apoplastic region of O_3_-exposed leaf cells. *AmO* amine oxidase, *AsA* ascorbic acid, *LEA-L* late embryogenesis abundant protein-like protein, *POD* cell-wall peroxidase, *Rboh* respiratory burst oxidase homolog.

Since at least two different O_3_-inducible responses, stomatal closure and visual foliar damage due to mesophyll cell death, can be suppressed in FOX-AtUC5 plants, AtUC5 is apparently involved in regulating the early steps in O_3_-induced responses that are shared by these two different physiological responses. Representative of such early responses are the production and subsequent signalling of ROS in the apoplastic region^22,29^ which may be the target of AtUC5 action. This hypothesis is supported by AtUC5 localization to the apoplast (Fig. 3c) and the difference in foliar content of TBARS, a ROS marker, between O_3_-treated FOX-AtUC5 and wild-type plants (Fig. 1c). As described at the beginning of this report, ROS can be generated by the direct reaction of O_3_ with either water or organic substances accompanied by its degradation in the apoplast. Additionally, considerable amounts of O_3_-induced ROS production in the apoplastic region are reportedly due to the activation of plasma membrane-bound respiratory burst oxidase homologs (NADPH oxidases) and cell wall peroxidases^18,30^. Moreover, other ROS-producing enzymes such as oxalate oxidase and amine oxidases are found in the apoplast, although they have not yet been reported to be involved in O_3_-induced responses^31^. The potential interaction of AtUC5 with copper amine oxidase indicated by yeast two-hybrid analysis suggests that the plastocyanin-like domain of AtUC5 may interact with the oxidase. Hydrogen peroxide produced by apoplastic amine oxidase have been reported to mediate hypersensitive cell death in virus-infected tobacco plants^32^ and abscisic acid-induced stomatal closure in *Vicia faba*^33^, respectively. Therefore, AtUC5 may inhibit O_3_-induced responses by suppressing ROS generation by copper amine oxidase (Fig. 6). Electron transfer between a phytocyanin and cell wall–localised oxidases, including amine oxidase, has been proposed to be involved in lignification of sclerenchymatous tissue in developing pea pods^34^.

Another possible interacting protein for AtUC5 suggested by yeast two-hybrid analysis is LEA protein-like protein. Although this protein’s physiological function is unknown, LEA proteins have been proposed to play an important role in abiotic stress resistance by either acting as a hydration buffer, sequestering ions, protecting other proteins or membranes directly, or by renaturing unfolded proteins^35,36^. Therefore, AtUC5 binding to an LEA protein-like protein suggests that the latter protects the former from inactivation or degradation under stress conditions. However, since some LEA proteins or LEA protein-like proteins reportedly have radical scavenging^37^ or antioxidative functions^38^, it would be interesting to investigate the function of the LEA protein-like protein that we identified in this study and its interaction with AtUC5 with respect to these functions. Furthermore, AtUC5 may interact with other proteins after post-translational modifications in plant cells occur, but not in yeast cells, such as plant-specific glycosylation. Alternatively, AtUC5 may interact with non-proteinaceous substances such as ROS or other low molecular weight substances such as ascorbic acid that are involved in either redox response or signalling. These interactions cannot be detected by yeast two-hybrid analyses. Further studies are therefore necessary to identify the target substance(s) of AtUC5 as well as other phytocyanins.

Although large numbers of phytocyanin-encoding genes have been reported in plant genomes, little information is available on the physiological function of most phytocyanins. However, some of them are reportedly involved in stress responses. The most extensively analysed phytocyanin is the *A. thaliana* stellacyanin AtSC3 (At5g20230) that has also been called “blue copper-binding protein (BCB)”. The gene encoding this protein was initially found among those genes induced by various stress factors such as aluminium and O_3_^39,40^. *AtSC3* expression has subsequently been shown to confer aluminium tolerance in yeast^41^ and tolerance to both aluminium and diamide in *A. thaliana*^42^. Since both aluminium and diamide are predicted to cause oxidative stress, AtSC3 function is potentially linked to the oxidative stress response. Furthermore, Ezaki et al.^43^ reported that transgenic *A. thaliana* overexpressing *AtSC3* showed enhanced lignin production and proposed that oxidative stress caused by aluminium may have been attenuated by enhanced consumption of ROS accompanying lignin synthesis in these plants. Phytocyanin involvement in regulating lignin synthesis has also been reported in other phenomena as described above. Lignins are major components of secondary cell walls and their synthesis is induced by various biotic and abiotic stress factors including O_3_, resulting in enhanced tolerance to some of the stress factors due to their barrier or antioxidant effects^44–46^. However, further studies are necessary to clarify the relationships between lignins or their metabolism and plant responses to O_3_ as well as other stress factors.

Our present study found that overexpression of a few members of phytocyanin, such as AtUC5 and AtSC3, conferred O_3_ tolerance in *A. thaliana*. Apart from fundamental physiological and biochemical studies, these phytocyanins are also good targets for developing O_3_-tolerant crops and trees for both agriculture and horticulture.

## Methods

### Plant material and growth conditions

Seeds from FOX lines were provided by RIKEN BRC through the National Bio-Resource Project of MEXT, Japan. Seeds of the *atuc5* (SALK_201823) knockout mutant were obtained from the Arabidopsis Biological Resource Center, Columbus, OH, USA. In addition, we generated various transgenic plants by *Agrobacterium*-mediated floral dip transformation of *A. thaliana* using the following vectors: pK2GW7 (Invitrogen, Gaithersburg, MD, USA) for *AtUC5-*overexpressing plants and *35S:GFP-AtUC5* plants; pK7FWG2 (Invitrogen) for *35S:AtUC5-GFP* plants; pBI121 (Clontech Laboratories, Inc., DE, USA) for *AtUC6-* and *AtSC3-*overexpressing plants. After transformation, a few plant lines with normal appearance and growth were selected for each DNA construct and their descendants that were homozygous for the transgenes were used for further analyses.

Plants from various lines were grown and exposed to O_3_ as described in Saji et al.^7,13^. For most experiments, seeds were sown on blocks of rock wool (M25S30, Nittobo, Tokyo, Japan) dipped in a nutrient solution (1/2000-strength Hyponex 6-10-5, Hyponex Japan, Osaka, Japan, containing two drops/L of HB-101, Flora, Yokkaichi, Japan) and the resultant seedlings were grown in a growth chamber at 25 °C under 60% relative humidity and 14 h of 100 µmol photons m^−2^ s^−1^ illumination per day by white fluorescent lamps.

### O_3_ treatment and analyses of stress responses

The O_3_ sensitivities of various plants were analysed using a variety of different methods and compared between different plant lines such as FOX-AtUC5 and wild-type plants. Two-week-old seedlings grown as above were transferred to a separate growth chamber and exposed to 0.3 µL L^−1^ O_3_ for 2–6 h at 25 °C under 70% relative humidity and continuous illumination by metal halide lamps (420 µmol photons m^−2^ s^−1^). O_3_ was produced by electrostatic discharge in dry O_2_ (model KS-2, Keisokuki Service Co., Tokyo, Japan) and monitored using a UV photometric O_3_ analyser (model EC9810 SERINUS10, Acoem Ecotech, Melbourne, Australia). Plant O_3_ sensitivity was measured by the degree of visual injury observed during O_3_ treatment or after a 1-day interval in the absence of O_3_ under 420 µmol photons m^−2^ s^−1^ illumination. In some experiments, O_3_ sensitivities were also measured as the amount of ion leakage from the leaves. Ion leakage, which indicates leakage of cell contents to bath water due to cell membrane damage, was measured by detaching the first and second leaves of three different plants at the petiole. The six leaves were combined and shaken in 1 mL of distilled water for 1 h, and an ion conductivity meter was used to measure electroconductivity of the bath water. The leaves and the remaining water were autoclaved, and the conductivity of the solution measured. Relative ion leakage was quantified by dividing the conductivity of the pre-autoclaved solution by that of the autoclaved solution. To investigate the degree of oxidative damage, the cellular content of thiobarbituric acid-reactive substances was quantified in extracts obtained from entire rosettes with BIOXYTECH MDA-586 ASSAY (OXIS International Inc., Tampa, FL, USA). Foliar stomatal conductance was measured using a leaf porometer (Decagon Devices Inc., Pullman, WA, USA).

### Genetic or biochemical analyses

The presence of transgenes in such plants as FOX lines and various other transgenic plants was determined by either seed germination on antibiotic-containing media or PCR using DNA extracts from plants. For seed germination, seeds were sown on half-strength Murashige and Skoog medium-agar plates containing either hygromycin B at 25 µg mL^−1^ (for FOX lines) or kanamycin at 50 µg mL^−1^ (for other transgenic plants). PCR was performed with either the primers shown in Supplementary Fig. 2c (for FOX lines) or for *AtUC5-*overexpressing plants, or primers with sequences corresponding to vector sequences, namely, forward (attB1): GGG GAC AAG TTT GTA CAA AAA AGC AGG CT and reverse (attB2): GGG GAC CAC TTT GTA CAA GAA AGC TGG GT.

To measure *AtUC5* mRNA levels, total RNA was extracted from entire rosettes using the RNeasy Plant Mini Kit (Qiagen, Tokyo, Japan) and cDNA was synthesised using ReverTra Ace qPCR RT Master Mix with gDNA Remover (TOYOBO, Osaka, Japan). Reverse transcription PCR was subsequently carried out using specific primers for *AtUC5* (forward: ATGTGTTTGCACCTGCCTCT, reverse: GCTGCTGGAACAATGACAGA), and for *ACTIN2* (forward: TAACCCAAAGGCCAACAGAG, reverse: TTCTCGATGGAAGAGCTGGT) as an internal control. Levels of mRNA encoding other phytocyanin genes were similarly quantified using primers for *AtUC6* (forward: CGGTTAAGACAGCGTTAGCA, reverse: CTGATGTTCCCAGGTCACAA) and *AtSC3* (forward: CGATGTTGGTGATGATACGG, reverse: TGGTCCAGTGGTGTTTAGCA).

Immunochemical detection of fusion proteins in *35S:GFP-AtUC5* and *35S:AtUC5-GFP* plants was carried out by western blotting against protein extracts from these plants using Simple Western™ (Bio-Techne, Minneapolis, MN, USA) and anti-GFP monoclonal antibody (GF200, Nacalai Tesque, Inc., Kyoto, Japan).

### Subcellular localization assay

Subcellular localisation of AtUC5 and GFP fusion proteins in *35S:GFP-AtUC5* and *35S:AtUC5-GFP* plants was determined using an inverted microscope (IX73, Olympus, Tokyo, Japan) equipped with a reflected fluorescence system (U-FGFP/IX3-FGFPXL, Olympus). Mature rosette leaves were excised from these plants for observation. Plasmolysis was induced by dipping leaves in 40% sucrose for 10–30 min before observation.

### Yeast two-hybrid analyses

Both yeast two-hybrid screens and pairwise interaction assays of candidate proteins were performed by Hybrigenics Services, S.A.S., Évry, France. The coding sequence of the mature AtUC5 (amino acids 25–152) was used as a bait to screen a random-primed *A. thaliana* seedlings cDNA library. Based on the first screen, 362 colonies were selected on medium lacking tryptophan, leucine, and histidine, supplemented with 2 mM 3-aminotriazole to limit bait autoactivation. Prey fragments of the positive clones were amplified by PCR and sequenced at their 5′ and 3′ junctions. The resulting sequences were used to identify corresponding interacting proteins in the GenBank database (NCBI). Among the positive clones extracted from this screening, there were fragments corresponding to amino acids 212–491 of copper amine oxidase, amino acids 195–337 of late embryogenesis abundant (LEA) protein-like, and amino acids 128–206 of extensin-2-like protein. We subsequently carried out the following pairwise interaction assays between mature AtUC5 and these proteins. Bait and prey constructs were transformed in the yeast haploid cells L40deltaGal4 and YHGX13 (Y187 *ade2-101::loxP-kanMX-loxP*), respectively. The diploid yeast cells were obtained using a mating protocol with both yeast strains. Various controls and interactions were tested in the form of streaks of three independent yeast clones on DO-2 and DO-3 selective media. The DO-2 selective medium lacking tryptophan and leucine was used as a control and to verify the presence of bait and prey plasmids. The DO-3 selective medium without tryptophan, leucine, and histidine was used to select for interaction between bait and prey.

### Statistical analyses

For quantitative analyses, the average and standard deviation of obtained data were calculated, and representative results from at least three independent experiments are shown in the figures. One-way analysis of variance (ANOVA) was used to determine the statistical significance among different samples. A probability level < 0.05 was considered statistically significant. A linear regression analysis was applied to investigate possible relationships between levels of transgene expression and O_3_ sensitivities (ion leakage from leaves) of the transgenic plants overexpressing various phytocyanin genes.

### Ethical approval

All methods were carried out in accordance with Cartagena protocol domestic law and related regulations and followed the instructions given by the providers of *Arabidopsis* seeds, RIKEN BRC through the National Bio-Resource Project of MEXT, Japan and the Arabidopsis Biological Resource Center, Columbus, OH, USA. The data presented in this study are available within the article and Supplementary Materials.

## Supplementary Information

Supplementary Information 1.

Supplementary Information 2.

## References

[1] Hough AM, Derwent RG (1990). Changes in the global concentration of tropospheric ozone due to human activities. Nature.

[2] Montes CM, Demler HJ, Li S, Martin DG, Ainsworth EA (2021). Approaches to investigate crop responses to ozone pollution: From O_3_-FACE to satellite-enabled modeling. Plant J..

[3] Pell EJ, Schlagnhaufer CD, Arteca RN (1997). Ozone-induced oxidative stress: Mechanisms of action and reaction. Physiol. Plant..

[4] Fiscus EL, Booker FL, Burkey KO (2005). Crop responses to ozone: Uptake, modes of action, carbon assimilation and partitioning. Plant Cell Environ..

[5] Kangasjärvi J, Jaspers P, Kollist H (2005). Signalling and cell death in ozone-exposed plants. Plant Cell Environ..

[6] Tamaoki M (2008). The role of phytohormone signaling in ozone-induced cell death in plants. Plant Signal. Behav..

[7] Saji S (2008). Disruption of a gene encoding C_4_-dicarboxylate transporter-like protein increases ozone sensitivity through deregulation of the stomatal response in *Arabidopsis thaliana*. Plant Cell Physiol..

[8] Vahisalu T (2008). SLAC1 is required for plant guard cell S-type anion channel function in stomatal signaling. Nature.

[9] Brosché M (2010). Natural variation in ozone sensitivity among *Arabidopsis thaliana* accessions and its relation to stomatal conductance. Plant Cell Environ..

[10] Monda K (2011). Environmental regulation of stomatal response in the *Arabidopsis* Cvi-0 ecotype. Planta.

[11] Conklin PL, Williams EH, Last RL (1996). Environmental stress sensitivity of an ascorbic acid-deficient Arabidopsis mutant. Proc. Natl. Acad. Sci. USA.

[12] Yoshida S (2006). Cytosolic dehydroascorbate reductase is important for ozone tolerance in *Arabidopsis thaliana*. Plant Cell Physiol..

[13] Saji S (2017). Ozone-sensitive Arabidopsis mutants with deficiencies in photorespiratory enzymes. Plant Cell Physiol..

[14] Overmyer K (2000). Ozone-sensitive Arabidopsis *rcd1* mutant reveals opposite roles for ethylene and jasmonate signaling pathways in regulating superoxide-dependent cell death. Plant Cell.

[15] Rao MV, Lee HI, Davis KR (2002). Ozone-induced ethylene production is dependent on salicylic acid, and both salicylic acid and ethylene act in concert to regulate ozone-induced cell death. Plant J..

[16] Kanna M (2003). Isolation of an ozone-sensitive and jasmonate-semi-insensitive *Arabidopsis* mutant (*oji1*). Plant Cell Physiol..

[17] Ahlfors R, Brosché M, Kollist H, Kangasjärvi J (2009). Nitric oxide modulates ozone-induced cell death, hormone biosynthesis and gene expression in *Arabidopsis thaliana*. Plant J..

[18] Joo JH, Wang S, Chen JG, Jones AM, Fedoroff NV (2005). Different signalling and cell death roles of heterotrimeric G protein alpha and beta subunits in the Arabidopsis oxidative stress response to ozone. Plant Cell.

[19] Samuel MA, Ellis BE (2002). Double Jeopardy: Both overexpression and suppression of a redox-activated plant mitogen-activated protein kinase render tobacco plants ozone sensitive. Plant Cell.

[20] Gomi K (2005). A mitogen-activated protein kinase NtMPK4 activated by SIPKK is required for jasmonic acid signaling and involved in ozone tolerance via stomatal movement in tobacco. Plant Cell Physiol..

[21] Yanagawa Y (2016). Mitogen-activated protein kinase 4-like carrying an MEY motif instead of a TXY motif is involved in ozone tolerance and regulation of stomatal closure in tobacco. J. Exp. Bot..

[22] Vainonen JP, Kangasjärvi J (2014). Plant signalling in acute ozone exposure. Plant Cell Environ..

[23] Castro B (2021). Stress-induced reactive oxygen species compartmentalization, perception and signalling. Nat. Plants.

[24] Ma H, Zhao H, Liu Z, Zhao J (2011). The phytocyanin gene family in rice (*Oryza sativa* L.): Genome-wide identification, classification and transcriptional analysis. PLoS One.

[25] Nersissian AM (1998). Uclacyanins, stellacyanins, and plantacyanins are distinct subfamilies of phytocyanins: Plant-specific mononuclear blue copper proteins. Protein Sci..

[26] Janero DR (1990). Malondialdehyde and thiobarbituric acid-reactivity as diagnostic indices of lipid peroxidation and peroxidative tissue injury. Free Radic. Biol. Med..

[27] Borner GHH, Lilley KS, Stevens TJ, Dupree P (2003). Identification of glycosylphosphatidylinositol-anchored proteins in Arabidopsis. A proteomic and genomic analysis. Plant Physiol..

[28] The Arabidopsis Information Resource (2022) Phoenix Bioinformatics. https://www.arabidopsis.org/index.jsp.

[29] Hasan MM (2021). Ozone induced stomatal regulations, MAPK and phytohormone signaling in plants. Int. J. Mol. Sci..

[30] Ranieri A, Castagna J, Pacini B, Baldan AMS, Soldatini GF (2003). Early production and scavenging of hydrogen peroxide in the apoplast of sunflower plants exposed to ozone. J. Exp. Bot..

[31] Cona A, Rea G, Angelini R, Federico R, Tavladoraki P (2006). Functions of amine oxidases in plant development and defence. Trends Plant Sci..

[32] Yoda H, Yamaguchi Y, Sano H (2003). Induction of hypersensitive cell death by hydrogen peroxide produced through polyamine degradation in tobacco plants. Plant Physiol..

[33] An Z, Jing W, Liu Y, Zhang W (2008). Hydrogen peroxide generated by copper amine oxidase is involved in abscisic acid-induced stomatal closure in *Vicia faba*. J. Exp. Bot..

[34] Drew JE, Gatehouse JA (1994). Isolation and characterization of a pea pod cDNA encoding a putative blue copper protein correlated with lignin deposition. J. Exp. Bot..

[35] Wise MJ, Tunnacliffe A (2004). POPP the question: What do LEA proteins do?. Trends Plant Sci..

[36] Tunnacliffe A, Wise MJ (2007). The continuing conundrum of the LEA proteins. Naturwissenschaften.

[37] Hara M, Fujinaga M, Kuboi T (2004). Radical scavenging activity and oxidative modification of citrus dehydrin. Plant Physiol. Biochem..

[38] Mowla SB (2006). Yeast complementation reveals a role for an *Arabidopsis thaliana* late embryogenesis abundant (LEA)-like protein in oxidative stress tolerance. Plant J..

[39] Richards KD, Schott EJ, Sharma YK, Davis KR, Gardner RC (1998). Aluminum induces oxidative stress genes in *Arabidopsis thaliana*. Plant Physiol..

[40] Miller JD, Arteca RN, Pell EJ (1999). Senescence-associated gene expression during ozone-induced leaf senescence in Arabidopsis. Plant Physiol..

[41] Ezaki B, Sivaguru M, Ezaki Y, Matsumoto H, Gardner RC (1999). Acquisition of aluminum tolerance in *Saccharomyces cerevisiae* by expression of the *BCB* or *NtGDI1* gene derived from plants. FEMS Microbiol. Lett..

[42] Ezaki B, Gardner RC, Ezaki Y, Matsumoto H (2000). Expression of aluminum-induced genes in transgenic *Arabidopsis* plants can ameliorate aluminum stress and/or oxidative stress. Plant Physiol..

[43] Ezaki B, Sasaki K, Matsumoto H, Nakashima S (2005). Functions of two genes in aluminium (Al) stress resistance: Repression of oxidative damage by the AtBCB gene and promotion of efflux of Al ions by the *NtGDI1* gene. J. Exp. Bot..

[44] Cabané M (2004). Condensed lignins are synthesized in poplar leaves exposed to ozone. Plant Physiol..

[45] Underwood W (2012). The plant cell wall: A dynamic barrier against pathogen invasion. Front. Plant Sci..

[46] Liu Q, Luo L, Zheng L (2018). Lignins: Biosynthesis and biological functions in plants. Int. J. Mol. Sci..

