# Supplementary material for: Phytocyanin-encoding genes confer enhanced ozone tolerance in Arabidopsis thaliana
Source: Sci Rep. 2022 Dec 22;12:21204. doi: 10.1038/s41598-022-25706-0 (PMC9780206; doi:10.1038/s41598-022-25706-0)
Supplement: Supplementary file 1 — Supplementary Information 1. [file 41598_2022_25706_MOESM1_ESM.pptx]

## Slide 1
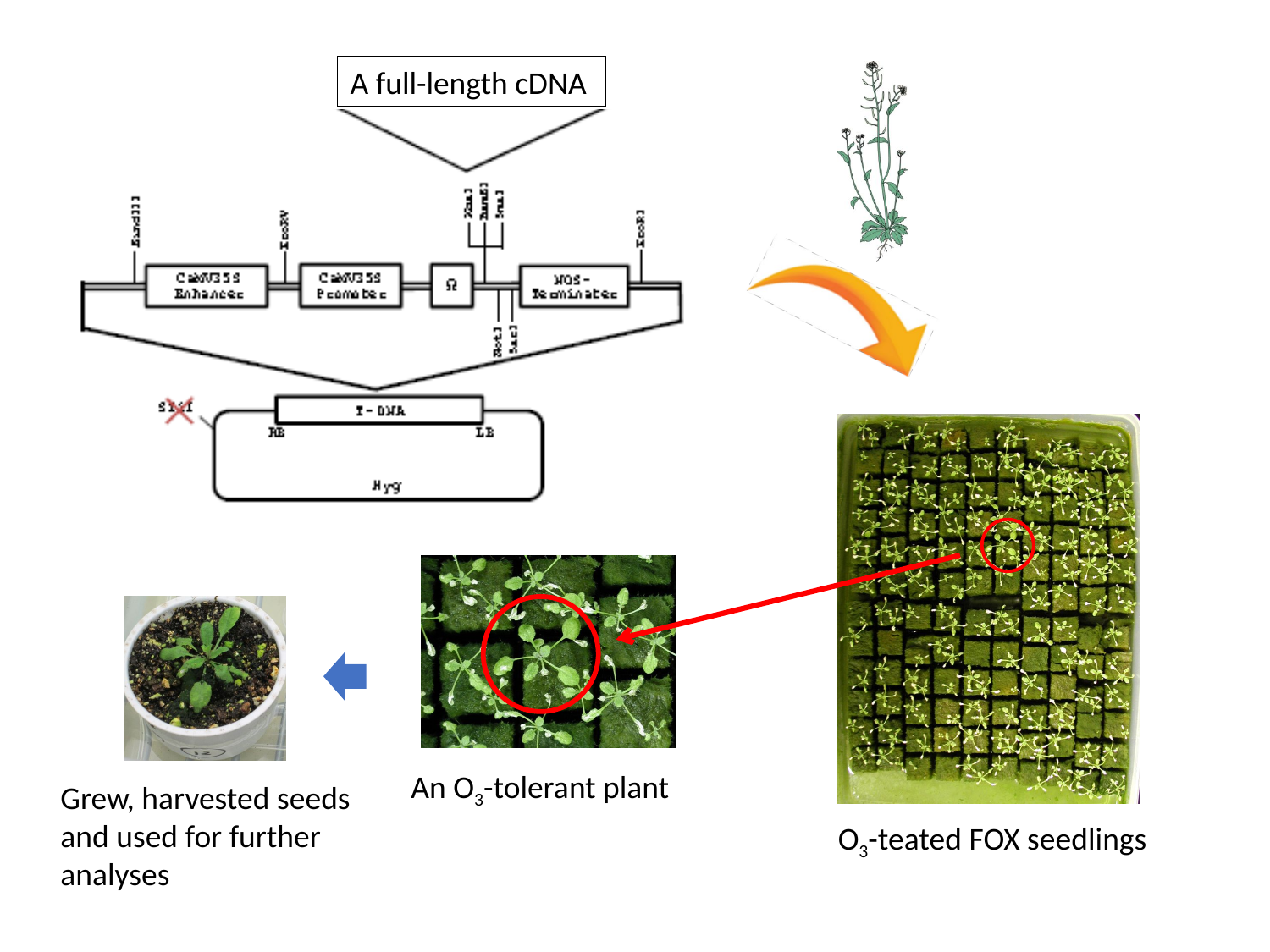

A full-length cDNA
An O3-tolerant plant
Grew, harvested seeds and used for further analyses
O3-teated FOX seedlings

## Slide 2
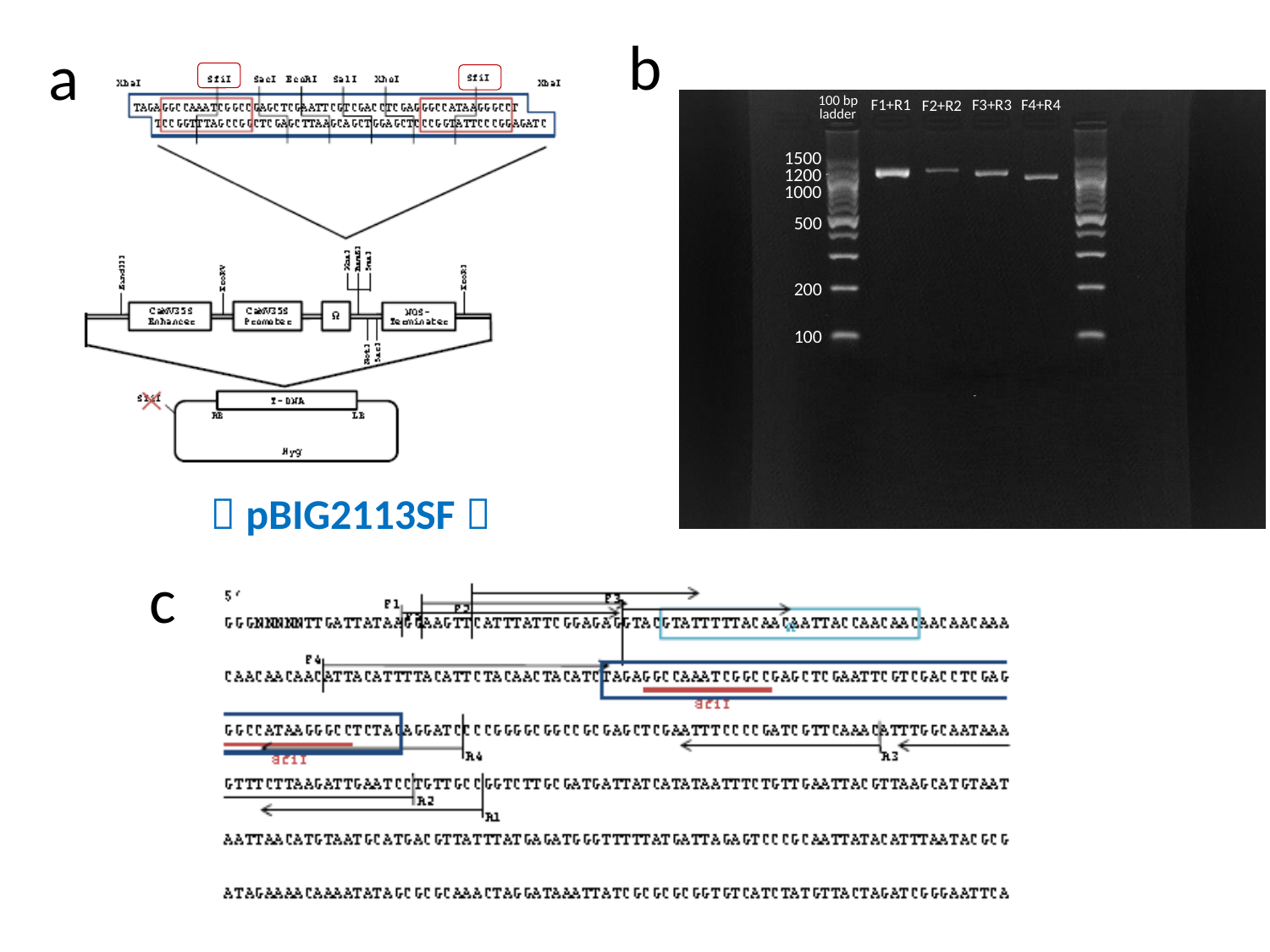

b
a
100 bp
F3+R3
F1+R1
F4+R4
F2+R2
ladder
1500
1200
1000
500
200
100
（pBIG2113SF）
c

## Slide 3
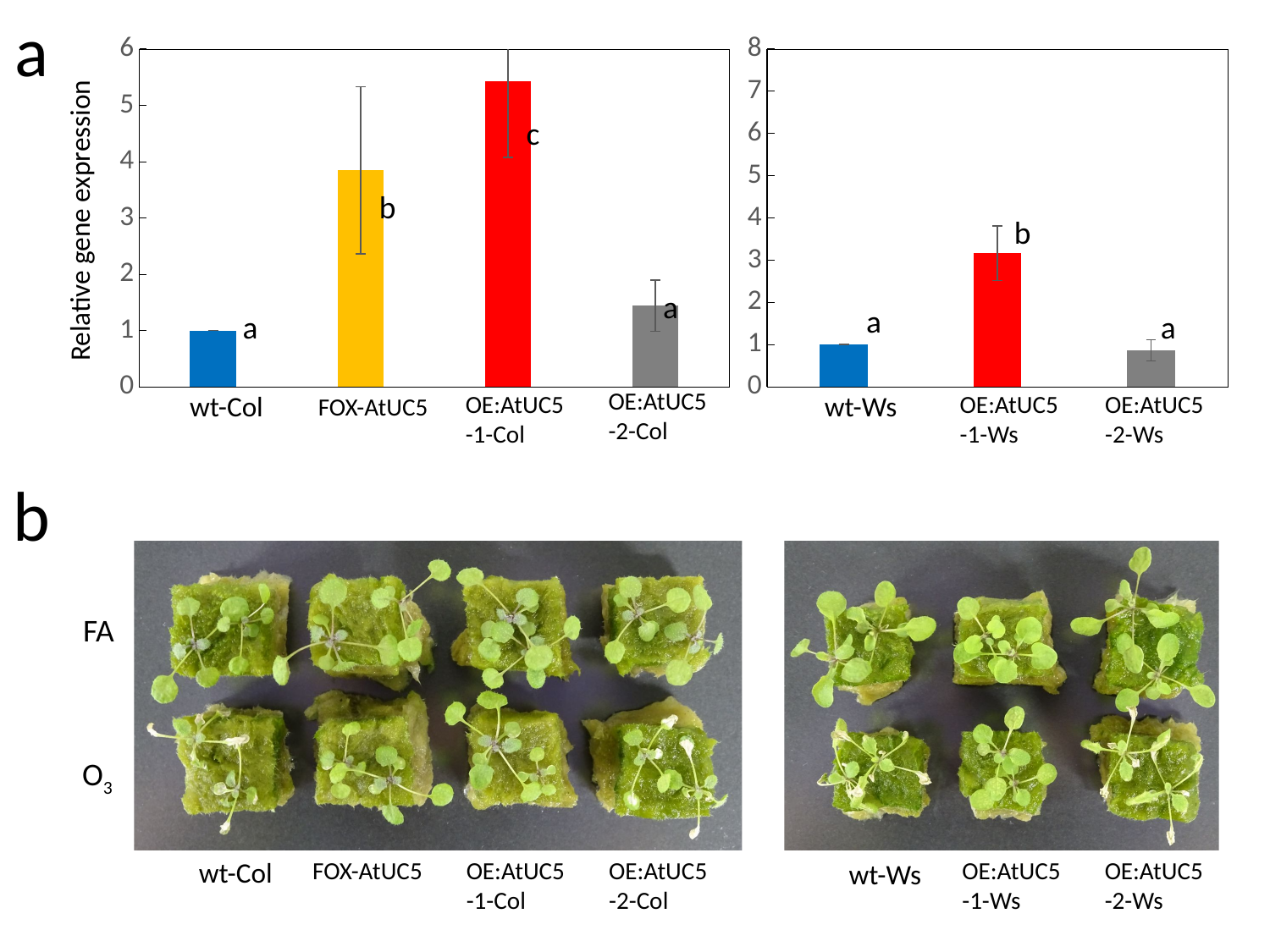

a
### Chart
| Category | |
|---|---|
| Col-0 | 1.0 |
| FOX | 3.8455515448885955 |
| o/e Col-0 O3r | 5.426197313811433 |
| o/e Col-0 O3s | 1.4406173933525654 |
### Chart
| Category | |
|---|---|
| Ws-2 | 1.0 |
| o/e Ws-2 O3r | 3.166863382761541 |
| o/e Ws-2 O3s | 0.8594521329781285 |c
b
Relative gene expression
b
a
a
a
a
OE:AtUC5
-2-Col
OE:AtUC5
-2-Ws
OE:AtUC5
-1-Ws
wt-Ws
wt-Col
OE:AtUC5
-1-Col
FOX-AtUC5
b
FA
 O3
FOX-AtUC5
wt-Col
OE:AtUC5
-1-Col
OE:AtUC5
-2-Col
OE:AtUC5
-1-Ws
OE:AtUC5
-2-Ws
wt-Ws

## Slide 4
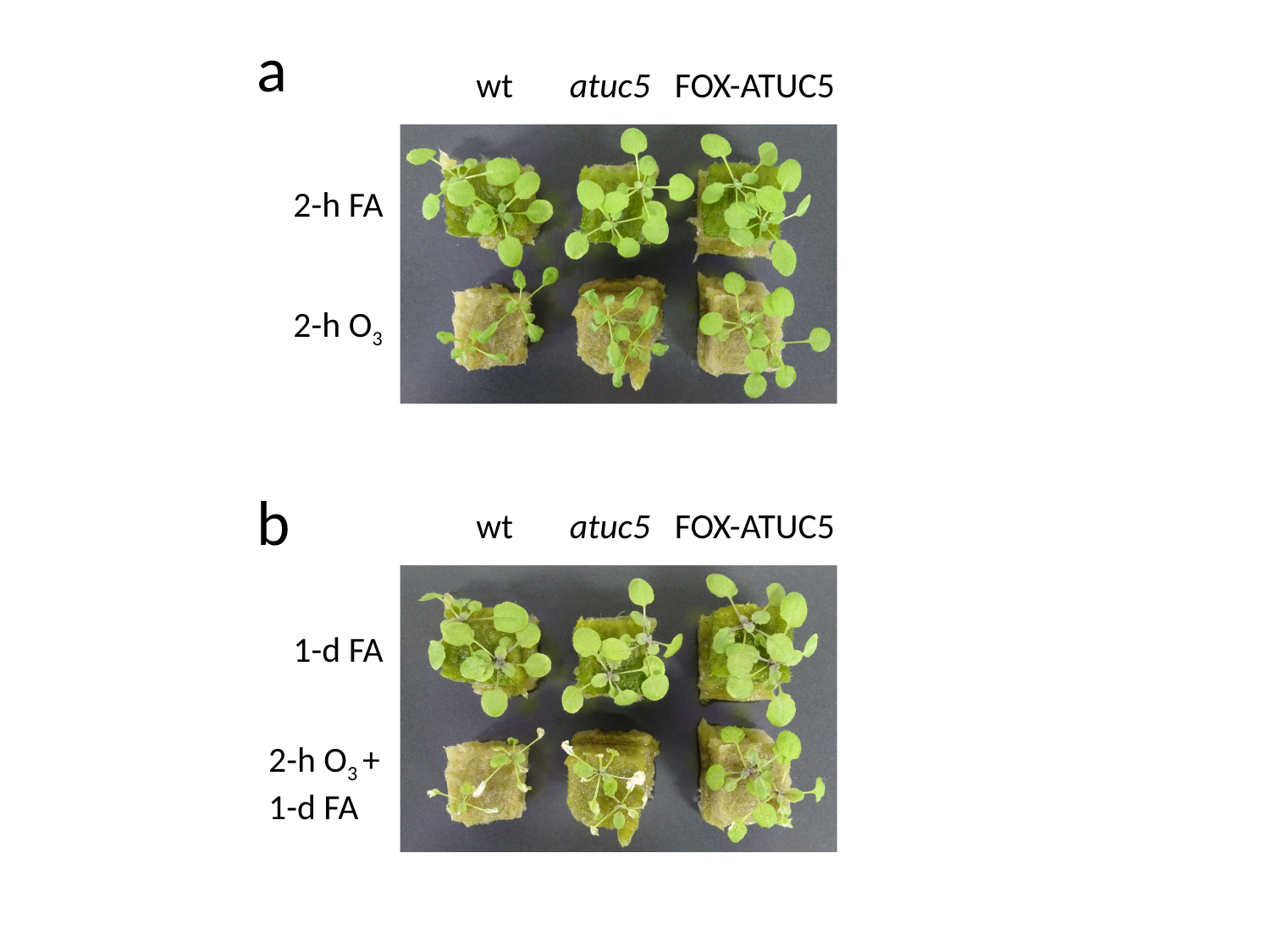

a
wt atuc5 FOX-ATUC5
2-h FA
2-h O3
b
wt atuc5 FOX-ATUC5
1-d FA
2-h O3 + 1-d FA
