# Supplementary material for: Phytocyanin-encoding genes confer enhanced ozone tolerance in Arabidopsis thaliana
Source: Sci Rep. 2022 Dec 22;12:21204. doi: 10.1038/s41598-022-25706-0 (PMC9780206; doi:10.1038/s41598-022-25706-0)
Supplement: Supplementary file 2 — Supplementary Information 2. [file 41598_2022_25706_MOESM2_ESM.pdf]

DomSight: ATH_RP3_hgx5609v1 vs. Arabidopsis thaliana seedlings_RP3 (09 Sep 2020)

(Bait plasmid(s): hgx5609v1_pB27)

Colour key	
Bait fragment	*SID: Selected Interaction Domain*
SID fragment	*It is the amino acid sequence shared by all prey fragments matching*
Pfam or SMART domain	*the same reference protein. SIDs have been found in numerous cases*
Transmembrane domain (TMHMM, Phobius)	*to correspond to an identified structural or functional domain.*
Coiled-coil domain (Ncoils)	*GenMatch/NoMatch SIDs are shortened at the first in-frame stop codon.*
Signal peptide (SignalP, Phobius)	*Protein Display:*
Cytoplasmic domain (Phobius)	*Proteins larger than 1500 aa are shown in sections of 1499 aa each.*
Non cytoplasmic domain (Phobius)	*Only sections containing bait fragments, SIDs or predicted functional and structural domains are represented.*

Legend

AT1G72230 — Arabidopsis thaliana, ACCESSION: NM_105882.3, Gene ID: 843555, AT1G72230 (1–181)
- 25 – 152 hgx5609v1
- 7 – 29 Transmembrane domain (tmhmm)
- 1 – 24 Signal peptide domain (phobius)
- 1 – 20 Signal peptide domain (signalp)
- 1 – 25 Signal peptide domain (signalp)
- 25 – 181 Non Cytoplasmic domain (phobius)
- 31 – 111 Phytocyanin domain - PF02298

D **ACO1** — Arabidopsis thaliana, ACCESSION: NM_127517.5, Gene ID: 816478, AT2G19590 (1–310)
- 5 – 230 SID
- 11 – 100 Non-haem dioxygenase N-terminal domain - PF14226
- 165 – 259 Oxoglutarate/iron-dependent dioxygenase - PF03171

D **ASN1** — Arabidopsis thaliana, ACCESSION: NM_180332.1, Gene ID: 823888 (1–512)
- 70 – 209 SID
- 1 – 16 Signal peptide domain (phobius)
- 17 – 512 Non Cytoplasmic domain (phobius)
- 48 – 165 Glutamine amidotransferase type 2 domain - PF13537
- 210 – 361 Asparagine synthase - PF00733

D **CDA1** — Arabidopsis thaliana, ACCESSION: NM_127515.4, Gene ID: 816476, AT2G19570 (1–301)
- 233 – 265 SID
- 34 – 115 Cytidine and deoxycytidylate deaminase d - PF00383
- 145 – 281 Cytidine/deoxycytidylate deaminase, zinc - PF08211

D **Copper amine oxidase family prot** — Arabidopsis thaliana, ACCESSION: NM_114235.4, Gene ID: 823469, AT3G43670 (1–687)
- 212 – 491 SID
- 5 – 24 Transmembrane domain (tmhmm)
- 1 – 24 Signal peptide domain (signalp)
- 1 – 24 Signal peptide domain (phobius)
- 25 – 687 Non Cytoplasmic domain (phobius)
- 41 – 123 Copper amine oxidase, N2-terminal - PF02727
- 133 – 231 Copper amine oxidase, N3-terminal - PF02728
- 257 – 676 Copper amine oxidase, catalytic domain - PF01179

D **DEHYDRATASE-ENOLASE-PHOSPH** — Arabidopsis thaliana, ACCESSION: NM_001345068.1, Gene ID: 835466, AT5G53850 (1–418)
- 1 – 129 SID
- 1 – 140 Class II aldolase/adducin N-terminal - SM01007
- 27 – 139 Class II aldolase/adducin N-terminal - PF00596
- 178 – 389 haloacid dehalogenase-like hydrolase - PF00702

D **F14D16.26** — Arabidopsis thaliana, ACCESSION: NM_101769.4, Gene ID: 838494, AT1G19110 (1–754)
- 54 – 205 SID
- 326 – 506 von Willebrand factor, type A - SM00327
- 328 – 485 von Willebrand factor, type A - PF13768

D **F16G20.240** — Arabidopsis thaliana, ACCESSION: NM_118485.9, Gene ID: 828454, AT4G23540 (1–1131)
- 135 – 480 SID
- 408 – 606 Uncharacterised domain NUC173 - PF08161

D **HSC70-1** — Arabidopsis thaliana, ACCESSION: NM_120328.5, Gene ID: 831020, AT5G02500 (1–651)
- 421 – 509 SID
- 521 – 541 Coiled domain
- 9 – 618 Heat shock protein 70 family - PF00012

B **IPMI1** — Arabidopsis thaliana, ACCESSION: NM_115761.3, Gene ID: 825068, AT3G58990 (1–253)
- 86 – 253 SID
- 135 – 187 Aconitase A/isopropylmalate dehydratase - PF00694

D **MDK4.20** — Arabidopsis thaliana, ACCESSION: NM_124817.5, Gene ID: 835525, AT5G54370 (1–337)
- 195 – 337 SID
- 7 – 29 Transmembrane domain (tmhmm)
- 1 – 24 Signal peptide domain (phobius)
- 1 – 24 Signal peptide domain (signalp)
- 1 – 24 Signal peptide domain (signalp)
- 25 – 337 Non Cytoplasmic domain (phobius)
- 246 – 302 Root cap - PF06830

D **PAP3** — Arabidopsis thaliana, ACCESSION: NM_001084069.1, Gene ID: 838035 (1–364)
- 122 – 364 SID
- 15 – 34 Transmembrane domain (tmhmm)
- 15 – 34 Transmembrane domain (phobius)
- 41 – 59 Transmembrane domain (phobius)
- 41 – 59 Transmembrane domain (tmhmm)
- 35 – 40 Cytoplasmic domain (phobius)
- 1 – 14 Non Cytoplasmic domain (phobius)
- 60 – 364 Non Cytoplasmic domain (phobius)
- 75 – 282 Calcineurin-like phosphoesterase domain, - PF00149

D **PAT2** — Arabidopsis thaliana, ACCESSION: NM_202714.4, Gene ID: 824714, AT3G55480 (1–1115)
- 698 – 980 SID
- 346 – 366 Transmembrane domain (phobius)
- 387 – 407 Transmembrane domain (phobius)
- 367 – 386 Cytoplasmic domain (phobius)
- 1 – 345 Non Cytoplasmic domain (phobius)
- 408 – 1115 Non Cytoplasmic domain (phobius)
- 40 – 639 Clathrin/coatomer adaptor, adaptin-like, - PF01602
- 795 – 947 AP-3 complex subunit beta, C-terminal do - SM01355
- 825 – 901 AP-3 complex subunit beta, C-terminal do - PF14796

D **PP2-A5** — Arabidopsis thaliana, ACCESSION: NM_105213.4, Gene ID: 842851, AT1G65390 (1–411)
- 205 – 377 SID
- 21 – 159 Toll/interleukin-1 receptor homology (TI - SM00255
- 23 – 192 Toll/interleukin-1 receptor homology (TI - PF01582
- 258 – 409 Phloem protein 2-like - PF14299

D **SDR4** — Arabidopsis thaliana, ACCESSION: NM_001339015.1, Gene ID: 822580, AT3G29250 (1–296)
- 38 – 151 SID
- 51 – 289 Enoyl-(Acyl carrier protein) reductase - PF13561

D **SYTA** — Arabidopsis thaliana, ACCESSION: NP_001189566.1, Gene ID: 816633, AT2G20990 (1–579)
- 327 – 485 SID
- 7 – 32 Transmembrane domain (phobius)
- 7 – 29 Transmembrane domain (tmhmm)
- 1 – 6 Cytoplasmic domain (phobius)
- 33 – 579 Non Cytoplasmic domain (phobius)
- 67 – 227 Synaptotagmin, SMP domain - PF17047
- 298 – 395 C2 domain - PF00168
- 299 – 399 C2 domain - SM00239
- 459 – 558 C2 domain - SM00239
- 459 – 561 C2 domain - PF00168

D **UBP12** — Arabidopsis thaliana, ACCESSION: NM_180452.1, Gene ID: 830548 (1–1115)
- 999 – 1115 SID
- 536 – 559 Coiled domain
- 55 – 159 MATH/TRAF domain - SM00061
- 60 – 180 MATH/TRAF domain - PF00917
- 198 – 520 Peptidase C19, ubiquitin carboxyl-termin - PF00443
- 624 – 874 Ubiquitin carboxyl-terminal hydrolase 7, - PF12436
- 884 – 1094 Ubiquitin carboxyl-terminal hydrolase, C - PF14533

D **UBQ10** — Arabidopsis thaliana, ACCESSION: NM_178968.5, Gene ID: 825880, AT4G05320 (1–457)
- 100 – 188 SID
- 1 – 72 Ubiquitin-like domain - SM00213
- 3 – 74 Ubiquitin-like domain - PF00240
- 77 – 148 Ubiquitin-like domain - SM00213
- 79 – 150 Ubiquitin-like domain - PF00240
- 153 – 224 Ubiquitin-like domain - SM00213
- 155 – 226 Ubiquitin-like domain - PF00240
- 229 – 300 Ubiquitin-like domain - SM00213
- 231 – 302 Ubiquitin-like domain - PF00240
- 305 – 376 Ubiquitin-like domain - SM00213
- 307 – 378 Ubiquitin-like domain - PF00240
- 381 – 452 Ubiquitin-like domain - SM00213
- 383 – 454 Ubiquitin-like domain - PF00240

D **UXS6** — Arabidopsis thaliana, ACCESSION: NM_128436.5, Gene ID: 817426, AT2G28760 (1–343)
- 1 – 115 SID
- 34 – 328 NAD(P)-binding domain - PF16363

D **phosphoglycerate mutase, 2,3-bis** — Arabidopsis thaliana, ACCESSION: NP_850542.1, Gene ID: 820006, AT3G08590 (1–560)
- 124 – 202 SID
- 21 – 544 Metalloenzyme - PF01676
- 103 – 323 BPG-independent PGAM, N-terminal - PF06415

D **transmembrane protein** — Arabidopsis thaliana, ACCESSION: NM_001334159.1, Gene ID: 842746, AT1G64385 (1–351)
- 16 – 180 SID
- 243 – 266 Transmembrane domain (phobius)
- 244 – 266 Transmembrane domain (tmhmm)
- 1 – 23 Signal peptide domain (signalp)
- 1 – 23 Signal peptide domain (phobius)
- 267 – 351 Cytoplasmic domain (phobius)
- 24 – 242 Non Cytoplasmic domain (phobius)

D **GenMatch** — Arabidopsis thaliana, ACCESSION: LR782546.1 (1–213)
- 1 – 12 SID

DomSight: ATH_RP3_hgx5609v1 vs. Arabidopsis thaliana seedlings_RP3 (09 Sep 2020)

(Bait plasmid(s): hgx5609v1_pB27)

Legend		
Bait fragment	*SID fragment*	
Bait fragment		***SID: Selected Interaction Domain***
SID fragment		*It is the amino acid sequence shared by all prey fragments matching the same reference protein. SIDs have been found in numerous cases to correspond to an identified structural or functional domain. GenMatch/NoMatch SIDs are shortened at the first in-frame stop codon.*
Pfam or SMART domain		***Protein Display:***
Transmembrane domain (TMHMM, Phobius)		*Proteins larger than 1500 aa are shown in sections of 1499 aa each. Only sections containing bait fragments, SIDs or predicted functional and structural domains are represented.*
Coiled-coil domain (Ncoils)		
Signal peptide (SignalP, Phobius)		
Cytoplasmic domain (Phobius)		
Non cytoplasmic domain (Phobius)		

Legend

B GenMatch
Arabidopsis thaliana
ACCESSION: LR782543.1
1 207
131 164 SID

C GenMatch
Arabidopsis thaliana
ACCESSION: LR699761.1
1 144
1 53 SID

A GenMatch
Arabidopsis thaliana
ACCESSION: LR782543.1
1 324
109 115 SID
195 200 SID
217 223 SID
1 22 Signal peptide domain (phobius)
1 22 Signal peptide domain (signalp)
23 324 Non Cytoplasmic domain (phobius)

A GenMatch
Arabidopsis thaliana
ACCESSION: LR782543.1
1 138
38 57 SID

D GenMatch
Arabidopsis thaliana
ACCESSION: LR782543.1
1 215
1 121 SID

B GenMatch
Arabidopsis thaliana
ACCESSION: LR782543.1
1 121
69 103 SID
